# Intestinal Infection of *Candida albicans*: Preventing the Formation of Biofilm *by C. albicans* and Protecting the Intestinal Epithelial Barrier

**DOI:** 10.3389/fmicb.2021.783010

**Published:** 2022-02-02

**Authors:** Ziyao Peng, Jianguo Tang

**Affiliations:** Department of Trauma-Emergency and Critical Care Medicine, Shanghai Fifth People’s Hospital, Fudan University, Shanghai, China

**Keywords:** *Candida albicans*, antibiofilm, intestinal epithelial barrier, new combination therapy, fungal infection

## Abstract

The large mortality and morbidity rate of *C. albicans* infections is a crucial problem in medical mycology. Because the generation of biofilms and drug resistance are growing concerns, the growth of novel antifungal agents and the looking for newer objectives are necessary. In this review, inhibitors of *C. albicans* biofilm generation and molecular mechanisms of intestinal epithelial barrier protection are elucidated. Recent studies on various transcription elements; quorum-sensing molecules; host responses to adherence; and changes in efflux pumps, enzymes, bud to hyphal transition, and lipid profiles have increased the knowledge of the intricate mechanisms underlying biofilm resistance. In addition, the growth of novel biomaterials with anti-adhesive nature, natural products, drugs, bioactive compounds, proteins, lipids, and carbohydrates are being researched. Recently, more and more attention has been given to various metal nanoparticles that have also appeared as antibiofilm agents in *C. albicans*. The intestinal epithelial obstacle exerts an crucial effect on keeping intestinal homeostasis and is increasingly associated with various disorders associated with the intestine such as inflammatory bowel disease (IBD), irritable bowel syndrome, metabolic syndrome, allergies, hepatic inflammation, septic shock, etc. However, whether their involvement in the prevention of other intestinal disorders like IBD are useful in *C. albicans* remains unknown. Further studies must be carried out in order to validate their inhibition functions in intestinal *C. albicans*. This provides innovates ideas for intestinal *C. albicans* treatment.

## Introduction

Fungal infections due to *Candida* species represent an important cause of nosocomial bloodstream infections and are especially usual among seriously ill and intensive care patients and the patients with a solid malignancy or in recovery from abdominal operation (Pongrácz et al., 2015; Li et al., 2016). As a normal element of human intestinal, oral, and vaginal microflora, *C. albicans* (*C. albicans*) is also the leading element causing nosocomial fungemia (Brown et al., 2012). Among several susceptible people, it is argued that *C. albicans* infections are disseminated by gastrointestinal transmission; data from researches applying both patients and animal models supports this assumption (Miranda et al., 2009; Maraki et al., 2015; Shankar et al., 2015). Life-threatening illness with obvious rates of mortality among immunocompromised patients and the patients undergoing immunosuppressive therapy can be caused by *C. albicans* (Pongrácz et al., 2015). *C. albicans* is a commensal fungus that asymptomatically adapts to the normal microflora of the host but is aggressive and virulent once transformed to its hyphal form and covered by an extracellular polymeric substance (EPS). This demonstrates that *C. albicans* is the most common fungal pathogen among humans. It can cause diseases varying from considerable mucosal infection to deadly invasive bloodstream infection, which has a 40% mortality rate (Gulati and Nobile, 2016; Lohse et al., 2018). The three phases of the growth of *C. albicans* include adherence of the yeast cells to medical equipment (early phase), differentiation of yeast cells and hyphal cells (intermediate phase), and a growth in the matrix, which is the mature stage (Alim et al., 2018).

Greatly structured biofilms consist of various cell kinds (i.e., round, budding yeast-form cells; oval pseudohyphal cells; and elongated, cylindrical hyphal cells) encased in an extracellular matrix (Chandra et al., 2001; Ramage et al., 2005, 2009; Fox and Nobile, 2012). Occupying 15% of all hospital-obtained sepsis cases, species within the CTG clade (mainly *C. albicans* and closely associated species) are the remarkable fungal species found in medical device infections as the fourth most usual cause of bloodstream infections in clinical context (Wenzel, 1995; Wisplinghoff et al., 2004; Pfaller and Diekema, 2007). Urinary and central venous catheters, mechanical heart valves, pacemakers, contact lenses, joint prostheses, and dentures are all impressionable to *C. albicans* biofilms (Donlan and Costerton, 2002; Kojic and Darouiche, 2004; Cauda, 2009; Seddiki et al., 2013). Once it generates on an implanted medical equipment, *Candida* biofilm is potential in seeding impregnated bloodstream infections and can result in aggressive systemic tissue and organ infections. Biofilms cause various infections by attaching to surfaces or interfaces and embedding in a matrix of extracellular polymeric substances (Costerton et al., 1999). Biofilm generation on biomaterials and medical equipment including catheters and heart valves, results in chronic infections with high morbidity and mortality rates (Uppuluri et al., 2010; Seddiki et al., 2013). Biofilms are remarkably resistant to drugs after implant-associated infections because matured biofilms form a protein- and carbohydrate-rich extracellular matrix. In virtue of the large resistance of fungal biofilms to present antifungal drugs, great antifungal doses and elimination of the colonized medical equipment are necessary for treating infections (Mermel et al., 2009; Lepak and Andes, 2011; Andes et al., 2012; Cornely et al., 2012; Lortholary et al., 2012). Therefore, novel antifungal agents are needed to stop biofilm generation (Zarnowski et al., 2014).

At the same time, intestinal epithelial cells (IECs) form the first physical and immunological protective wall against aggressive pathogens. They not only coexist with the intestinal microbiota resisting commensal bacteria, but also fight pathogens to maintain homeostasis (Sánchez de Medina et al., 2013). The intestinal mucosal obstacle is chiefly composed of a mechanical obstacle, a chemical obstacle, a microbial obstacle, and an immune obstacle, which exert a significant effect on blocking the attaching of pathogens including *Candida* species (Yan et al., 2013). The mucosal barrier can adequately contain luminal microorganisms and molecules while absorbing nutrients. Alterations of the intestinal epithelial barrier are increasingly being associated with various disorders related to the intestine such as irritable bowel syndrome, inflammatory bowel disease (IBD), metabolic syndrome, hepatic inflammation, allergy, septic shock, and others (Natividad and Verdu, 2013). Several studies have documented that farnesol promotes intestinal epithelial barrier transcriptional regulation by activating JAK/STAT3 signaling. The involved molecules may also represent a good potential target for the treatment of *C. albicans* invasion (Fang et al., 2019). Recent studies have shown that labetalol decreased TBI-caused sympathetic hyperactivity, and restrained histopathological intestinal injury combined with variations in gut permeability and gut TNF-α expression in a rat model of TBI (Lang et al., 2015).

Because broad-spectrum antifungal drugs are extensively applied, the appearance of resistant fungal strains in clinical cases has been a main issue in antifungal therapy. Here, we reviewed current research progress in inhibiting *C. albicans* biofilm formation and summarized current elements of intestinal epithelial barrier protection. These treatments may alter traditional antifungal drugs so that it becomes a novel therapeutic solution for *C. albicans* intestinal infection.

## Antibiofilm Treatment

### Natural Products

Over the past several decades, natural compounds have become an important source of antimalarial, antibacterial, and chemotherapeutic agents. Currently, approximately 60% of drugs applied for treating cancers are obtained from natural sources. In addition, one of the most usual and productive methods of obtaining new therapeutic agents applying medicinal chemistry is to modify natural products (Zaki et al., 2019). Natural commodity screening has proven to be a hopeful strategy. Therefore, it is effective to target fungal biofilms with natural derivatives or synthetic analogs. Antibiofilm agents can make fungal biofilms more impressionable against traditional antibiotics and the hosts’ immune systems but might not directly kill the bacteria. The search for *C. albicans* inhibitors has resulted in the identification of many compounds of potential therapeutic use.

Eucarobustol E (EE), a currently reported formyl-phloroglucinol meroterpenoid, displayed potent inhibitory roles against both *C. albicans* yeast cells and biofilms, but no poisonousness toward human cells. Observing an obvious increase in negative regulator genes (TUP1, NRG1), researchers assumed that eucarobustol E’s suppression of carbon flow to ergosterol activated the mechanisms of negative hyphal development management and finally contributed to biofilm suppression *in vitro* model (Liu et al., 2017). The biofilm generation of *C. albicans* can be inhibited by CLEO, camphor, or fenchyl alcohol at 0.01% treatments. The treatments appear to prevent hyphal formation, which might be beneficial in controlling *C. albicans* infections *in vitro* (Manoharan et al., 2017). Several vitro studies have documented that purpurin suppressed *C. albicans* biofilm formation by blocking the yeast-to-hypha change under hypha-inducing conditions at sublethal concentration (3 mg/ml) and decreased the metabolic activity of mature biofilms in a way dependent of concentration (Tsang et al., 2012). Recently, researchers have tested 21 methylindoles and found that biofilm formation was effectively inhibited by 1-methylindole-2-carboxylic acid (5MI2CA) at 0.1 mM (17.5 μg\ml) and 5-methylindole-2-carboxylic acid (5MI2CA) at 0.1 mM with *C. albicans* DAY185 and ATCC10231 strains *in vitro* research (Lee et al., 2018). In another study, researchers investigated biofilm-inhibiting activity against *C. albicans* with used different indole derivatives. The vitro and vivo research showed that 7-benzyloxyindole, 4-fluoroindole and 5-iodoindole suppressed biofilm generation more effectively than antifungal agent fluconazole (FCZ). In particular, while reducing *C. albicans* biofilm formation,7-benzyloxyindole at 0.02 mM (4.5 lg m l μ1) did not have an additive effect on planktonic cells (Manoharan et al., 2018). A recently vitro and vivo study proposed a set of compounds, on basis of the Pseudomonas aeruginosa 2-heptyl-4(1H)-quinolone (HHQ) key quinolone interkingdom signal structure, that manifest non-cytotoxic antibiofilm activity in *C. albicans*’ fungal pathogens (Reen et al., 2016). Interestingly, an *in vitro* study showed that riccardin D as a macrocyclic bisbibenzyl separated from Chinese liverwort Dumortiera hirsute plays an inhibitory role in *C. albicans* biofilm generation (Li et al., 2012). In addition, as a novel kind of antifungal agent, bisbibenzyls suppress morphogenesis switches and biofilm generation by upregulating DPP3 in *C. albicans in vitro* (Zhang et al., 2011). Perillaldehyde (PAE), a natural monoterpenoid agent extracted from *Perilla frutescens*, has been proved to have multiple physiological capabilities, which is available as an anti-inflammatory, anti-oxidative and antifungal agent. Study has demonstrated that PAE manifests powerful antifungal ability against *C. albicans* (*C. albicans*). Researchers found that PAE prevented NLRP3 inflammasome assembly, decreased the extreme accumulation of ROS and inhibited the p65 transfer in nuclear; all resulting in decreased inflammation in the host. Together, these evidences suggest using PAE to treat *C. albicans* infection *in vitro* and vivo (Chen et al., 2020). Shikonin (SK) is the president component of the red pigment extracts from the roots of the plant Lithospermum erythrorhizon. It not only could prevent the formation of biofilms but also break the maintenance of mature biofilms. In a mouse vulvovaginal candidiasis (VVC) model, the fungal burden was largely decreased after SK treatment. Another studies demonstrated that SK is able to prevent hyphae formation and decrease cellular surface hydrophobicity (CSH). Some hypha-and adhesion-specific genes were distinguishingly expressed in SK-treated biofilm, containing the downregulation of ECE1, HWP1, EFG1, CPH1, RAS1, ALS1, ALS3, and CSH1 and upregulation of TUP1, NRG1, and BCR1 *in vitro*. Furthermore, SK could induce the production of farnesol, a quorum sensing molecule, and an exogenous addition of farnesol improved the antibiofilm activity of SK (Yan et al., 2019).

### Antifungal Agents

One challenge for clinicians is that there is a limited number of available antifungal agents. There are three classes of antifungals: azoles, polyenes, and echinocandins, which are primarily used for invasive infections. Although azoles including fluconazole, have been the major treatment method for *Candida* infections for nearly two decades, a decline in susceptibility to azoles, polyenes, and echinocandins by *Candida* species is an expanding problem. The limited quantity of antifungal drugs and the growth in resistance to present antifungals necessitate the discovery of some new antifungal agents (Vila et al., 2016). Presently, FCZ is the preferred therapy in systemic C. albicans infection (Parizkova et al., 1999). Recent research shows that a novel topical triazole PC945 has antifungal activity against emerging yeast Candida auris *in vitro* (Rudramurthy et al., 2019).

Miceli et al. (2009) have shown that doxycycline (DOX; 128 μg/mL) alone had similar role as FCZ (2–1,024 μg/mL) against *C. albicans* biofilms, which only result in a 22.9% reduction of biofilm metabolic activity. Nevertheless, when DOX (128 μg/mL) used in combination with FLC it had an important synergistic effect, resulting in that biofilm metabolic activity of the *C. albicans* biofilm was reduced 58.3%. Furthermore, when DOX was used alone at a higher concentration (2,048 μg/mL) it cause more significant effect, with a 85% increase in reduction. These results demonstrate that the combination of a high-dose DOX-based antimicrobial lock therapy and traditional antifungal agents may be more advantageous to the treatment of *C. albicans* biofilms *in vitro* (Miceli et al., 2009). In another vitro study, researchers have observed that the synergistic effects and mechanisms of the combination of FLC and DOX at a lower concentration (1–64 μg/mL) against *C. albicans* biofilms (Gao et al., 2013). Some vitro studies have reasearchedresearched the effects of NSAIDs on fungal growth inhibition, enzyme activation, and reduction in fungal prostaglandin E2 (PGE2) production, particularly focusing on inhibiting biofilm formation; For instance, aspirin could decrease the biofilm formation, with a 95% reduction. The inhibitory effects on *C. albicans* of COX inhibitors plus FLC block biofilm development through the PGE2-dependent mechanism, which suggests a new method of solving the biofilm resistance problem (Alem and Douglas, 2004; de Quadros et al., 2011; Ells et al., 2011). In addition, ibuprofen exhibited a synergistic effect with FLC against FLC-resistant strains (but not FLC-susceptible strains) *in vitro* (Arai et al., 2005; Ricardo et al., 2009). Vitro studies suggest that ambroxol (AMB) could easily penetrate the formed biofilm and exert antifungal effects, thereby blocking biofilm formation. The finding herein provide the first mode of action of the antifungal and antibiofilm activity of the mucolytic agent and its advantage to terbinafine as a commercially available antifungal that can prevent the fungal growth and biofilm formation (Rene et al., 2014). Researchers found that anidulafungin exerts an additive effect on immune cells which prevents *Candida* biofilms formation. Moreover, this additive interaction contributes to the release of the proinflammatory cytokine TNF-α and the chemokine IL-8 at different levels. The helpful Th1 response observed after therapy of biofilms with anidulafungin could provide new therapeutic ideas, including inhibiting the release of cytokines with harmful effects and the induction of others with beneficial effects *in vitro* (Katragkou et al., 2010). Besides, D,L-2-hydroxyisocaproic acid (HICA) may become a promising antifungal agent to prevent *C. albicans* cell growth and biofilm formation due to abnormal hyphae and collapsed hyphal structures in time of incubation with HICA at an acidic pH. It’s necessary for treating bacterial-fungal biofilm infections to importantly decrease the mutagenic potential of *C. albicans* biofilms *in vitro* (Nieminen et al., 2014). Clioquinol [5-chloro-7-iodoquinolin-8-ol, (CQ)] manifested fungistatic and fungicidal activity against *C. albicans*. It blocked true hyphae formation in a way of concentration-dependent in a variety hyphae-inducing of conditions. CQ also has interferential effect on ion homeostasis in *C. albicans* to prevent the growth of fungi in the vitro model, which is different from the present antifungal agents (You et al., 2020). Farnesol as the first quorum-sensing molecule was discovered in a eukaryote. It prevented the development of biofilms formed by the resistant strain. Moreover, there were synergistic effects between farnesol and fluconazole/5-fluorocytosine, but there were antagonistic effects between farnesol and terbinafine/itraconazole, respectively, on the biofilms formed by the resistant strains *in vitro* (Xia et al., 2017).

The new bacterial quorum sensing quencher thiazolidinedione-8 (S-8) possessed given antibiofilm and antiadhesion activities against *C. albicans*. The expression extents of genes correlated with biofilm generation, adherence, and filamentation (HWP1, ALS3, and EAP1, respectively) were downregulated by S-8 dose-dependently. Therefore, S-8 presents a new antibiofilm therapeutic approach in treating and preventing biofilm-related *C. albicans* infections *in vitro* model (Feldman et al., 2014). Recently vitro and vivo studies revealed that phenylthiazole small molecules including compound 1, have become an important research subject as novel antifungal agents for drug-resistant *Candida* infections. These molecules manifest rapid fungicidal activity and reduce the metabolic activity of appendiculate *C. albicans* and *C. auris* biofilms by more than 66 and 50%, respectively (Mohammad et al., 2019). CD101 is a long-acting novel echinocandin with unique pharmacokinetic characteristics and effective stability and safety relative to the same drug class, acting powerfully against early and mature *C. albicans* biofilms *in vitro* (Chandra and Ghannoum, 2018). In addition, ionic liquids are a new category of molten salts. These compounds have been used as ingredients of active pharmaceutical ingredients and antimicrobials. Previous vitro studies have shown that imidazolium ionic liquid compounds have antifungal and antibiofilm activities by influencing different cellular processes (Reddy and Nancharaiah, 2020). In another study, oral management of the broad-spectrum antibiofilm compound toremifene suppresses *C. albicans* and staphylococcus aureus biofilm generation *in vivo*, exhibiting a promising possibility of toremifene use as a broad-spectrum oral antibiofilm compound (De Cremer et al., 2014). Nitric-oxide releasing aspirin (NO-ASA) has an antifungal/antibiofilm effect on *C. albicans* separates from denture stomatitis patients *in vitro*, which demonstrates NO-ASA’s potential as a novel antibiofilm agent for treating fluconazole-resistant strains of *C. albicans* (Madariaga-Venegas et al., 2017). Miltefosine is an alkylphosphocholine showing potent antiparasitic activity. This compound has been proven to inhibit *C. albicans* and non-*albicans Candida* spp. biofilms and impair the interspersion of infectious cells *in vitro* (Vila et al., 2016). Another new discovery has identified that caspofungin as an antifungal agent acted effectively against biofilms by intensely reducing biofilm dispersion under flow conditions *in vitro* (Uppuluri et al., 2011).

### Microorganisms

Microorganisms could synthesize different types of surface-active compounds that are effective as antifungal, antibacterial, anti-adhesive, and antibiofilm agents, which could make them useful as main immunomodulatory molecules or in vaccines and gene therapy (Barbosa et al., 2016).

A recent vitro study revealed that *Lactobacillus* strains acted against *Candida*, and the strains’ biosurfactants were anti-adhesive and impeded biofilm activity against *C. albicans* (dos Santos et al., 2019). Given that *lactobacilli* and *C. albicans* are present in all regions of the human GI tract, including the low-biodiversity niches of the stomach and small intestine, *Lactobacillus* species may be central to preventing the outgrowth of *C. albicans* and other similarly resilient opportunistic pathogens (Zeise et al., 2021). *In vitro* study, butyrate isolated from *Lactobacillus* cultures can inhibit *C. albicans* hyphal morphogenesis (Noverr and Huffnagle, 2004). Another group found that butyrate inhibited C. albicans growth and filamentation but also enhanced the production of nitric oxide by macrophages and thus their ability to kill C. albicans cells *in vitro*. While this group did not implement any experiments to directly link the effects of butyrate on C. albicans virulence to its function as a HDACi, they hypothesized that that was the likely mechanism (Nguyen et al., 2011). In addition, researchers show that some *Lactobacillus* species produce a small molecule under laboratory conditions that blocks the C. albicans yeast-to-filament transition, an important virulence trait. Bioassay-guided fractionation of Lactobacillus-conditioned medium linked this activity to 1-acetyl-β-carboline (1-ABC). They use genetic approaches to show that filamentation inhibition by 1-ABC requires Yak1, a DYRK1-family kinase. Additional biochemical characterization of structurally related 1-ethoxycarbonyl-β-carboline confirms that it inhibits Yak1 and blocks *C. albicans* biofilm formation. Thus, our findings reveal Lactobacillus-produced 1-ABC can prevent the yeast-to-filament transition in *C. albicans* through inhibition of Yak1 *in vitro* and vivo (MacAlpine et al., 2021). Graham et al. (2017) reported that recognition of the *E. faecalis* bacteriocin, EntV [generated from the entV (ef1097) locus], is essential and adequate for decreasing C. albicans virulence and biofilm generation by inhibiting hyphal generation *in vitro* and vivo research (Graham et al., 2017). Phagocytic cells are crucial components of the innate immune system preventing *C. albicans* mucosal infections. *Streptococcus gordonii* and *Pseudomonas aeruginosa* often colonize mucosal sites, along with *C. albicans*. *S. gordonii* increased *C. albicans* survival and filamentation within macrophage phagosomes, while *P. aeruginosa* reduced fungal survival and filamentation (Salvatori et al., 2020). In another vitro and vivo study, *S. mutans* were capable of secreting subproducts that inhibited biofilm generation, morphogenesis and pathogenicity in *C. albicans*, alleviating test candidiasis in the *G. mellonella* model (Barbosa et al., 2016).

### Phenotypic Screening

Phenotypic screening means the nascent methodology for biological screening of chemical entities for the assessment of their therapeutic roles. Phenotypic screening was useful for identifying various small molecules exhibiting antibiofilm and anti-filamentation activity against *C. albicans*. Recent studies have shown that a new range of diazaspiro-decane structural analogs were often elements of bioactive compounds, which prevent processes connected with *C. albicans* virulence (most remarkably biofilm generation and filamentation) without affecting overall growth or generating resistance *in vitro* and vivo (Pierce et al., 2015). One study leads to the identification of about 2,293 compounds from the chemical library of the National Cancer Institute which were categorized into three sets- (i) NCI Natural set, Out of all the compounds present in this set six hits were confirmed against *C. albicans* biofilm formation. These include -Trichoderonin; Nanaomycin; Rapamycin; Anisomycin; V alinomycin and Bacitracin. Three of these molecules (Trichoderonin, Nanaomycin, Rapamycin) showed inhibition of both filamentation and biofilm formation while the rest three showed inhibition against biofilm formation. Furthermore in the next (ii)-NCI-Structural diversity set, in total, there were 12 hits out of which eight were identified as biofilm inhibitor. These compounds were-Phenanthroline Hydrochloride; 2-isoquinolin-2-ium-2-yl-1-phenanthren-3-ylethanone, Iodide; Metanilamide (3-aminobenzenesul fonamide); Mercury, (4-amino phenyl) (6-thioguano sinato-N7,S6)-; 2-[7-[3-(carboxymethyl)-5,10-dihydroxy-1-methyl-6,9-dioxo-3,4-dihydro-1H-benzo[g] isochr omen-7-yl]-5,10-dihydroxy-1-methyl-6,9-dioxo-3,4-dihydro-1H -benzo [g]isochromen-3-yl]acetic acid 37 are only biofilm inhibitors while Mercury,(2-aminio-1,9-dihydro-6H-purine-6 -thionato-N7,S6) hexyl-,2-benzo[a]phenothiazin-12-yl-N,N diet hylethanamine; 17-[1-[2(dimethylamino)ethylamino]ethyl]-13-methyl-6,7,8, 9,11,12,14,15,16,17 decahydrocyclopenta[a]phen anthren-3-ol. Next and last was (iii)-NCI-Challenge Set. In this, there were total 11 hits, of which 10 showed inhibition against *C. albicans* biofilm formation whereas only one compound inhibited filamentation transformation. Ten hits which were identified from these compounds displayed common inhibition against both biofilm and filament formation. These include Biofilm Inhibitor- Trichopolyn-B, Vengicide (Unamycin B, Toyocamycin), 4Z-4-[[4-(dimethylamino)phenyl]methylidene]-1-methyl-2-phenylpyrazolo[1,5-a]indol-1-ium-6-ol; trifluorome thanesulfonate, Anisomycin43, Azetidinecarbo thioic acid, [1-(2-pyridinyl) ethylidene] hydrazide4. Additionally, compounds with both antifilamentation and antibiofilm activity are—6-Hydroxy-3-[(methanesulfonyloxy) Methyl]-1-[(5,6,7-tri methoxyindol-2-yl) carbonylindoline, Hydrazineca rbothioamide, N,N-dipropyl-2-(2-pyridinemethylene)-,(N,N,S) copper(II)chloridecomplex(SP-4-3)3;3-Azabicyclo[3.22]nonane-3-carboselenoicacid,[1-(2pyridinyl)ethyidene] hydrazide, 2-hyd roxyethyl-[(2R)-2-hydroxyheptadecyl]-dimethylazaniumiodide, 1H-Azepine-1-carbothioic acid, hexa hydro-, [1-(2-pyridinyl) ethylidene]hydrazide (Pierce et al., 2014).

### Protein and Peptide Inhibitors

Researchers are trying to develop effective and potent therapies designed to eradicate biofilm-associated infections. Among these, antimicrobial peptides (AMPs), cytokines, and various proteins have been examined extensively as new therapeutic agents. Repetitive Lysine-Tryptophan Peptide scans are capable of inhibiting cellular functions by binding to RNA and DNA after it has been translocated into the cell, contributing to the inhibition of biofilm formation in a fluconazole-resistant *C. albicans* strain and the eradication of *C. albicans* (Ramamourthy et al., 2020). Researchers selected four of peptides (cathelicidin-BF, Pc-CATH1, Cc-CATH2, Cc-CATH3) and human cathelicidin LL-37 to carefully examine their anti-*C. albicans* and antibiofilm activities *in vitro* and *in vivo*. Antimicrobial assay suggesting that Pc-CATH1, Cc-CATH2, Cc-CATH3 and cathelicidin-BF have valid antifungal activities against the eight tested *C. albicans* strains, containing standard and clinically isolated amphotericin B-resistant strains. Furthermore, cathelicidin-BF importantly prevented the formation of *C. albicans* biofilms at sub-antimicrobial concentrations, and also manifested powerful activity of killing *C. albicans* in preformed biofilms (Yu et al., 2016). In another study, the naturally happening host defense peptide, LL-37, and its truncated mimetics KE-18 and KR-12 were biocidal and antibiofilm against *C. albicans*, Escherichia coli and staphylococcus aureus *in vitro* (Luo et al., 2017). Adopting an *in vitro C. albicans* biofilm model, research suggests that TNF dose-dependently suppresses biofilm growth stopped by cultivating TNF with N,N′-diacetylchitobiose, a main carbohydrate ingredient of the *C. albicans*’ cell wall (Rocha et al., 2017). Weiland-Brauer et al. (2019) assessed the capacity of a multitude of metagenome-derived bacterial quorum quenching (QQ) proteins to block biofilm development in *C. albicans* and S. epidermidis. Here, proteins QQ-5 and QQ-7 obstructed the morphogenesis of *C. albicans* by suppressing a yeast-to-hyphae conversion and impairing biofilm formation *in vitro* model (Weiland-Brauer et al., 2019). Along with this, the hLF1-11 peptide notably inhibited *C. albicans* biofilm formation primarily at early stages, disturbing biofilm cellular density and metabolic activity, and influenced morphogenesis in the Ras1-cAMP-Efg1 path *in vitro* (Morici et al., 2016). By binding the most hopeful amino acid substitutions, researchers observed that the double-substituted OSIP108 analog Q6R/G7K displayed eight-fold-grown antibiofilm activities (Delattin et al., 2014).

### Lipid Inhibitors

Based cluded that cinnamaldehyde (CNMA) could potentially be used in multilamellar liposomes (ML) as an antifungal and antibiofilm agent. According to the outcomes of a vitro research, ML-CNMA blocks the proliferation of *C. albicans* and accelerates apoptosis (Khan et al., 2017). In another study, it has been proved that sophorolipid (SL), a glycolipid biosurfactant, has antimicrobial and anticancer characteristics. It was found that SL retards *C. albicans* biofilm formation and decreases the survival of conducted biofilms *in vitro* (Haque et al., 2016). Researchers have found that SMOFlipid did not damage *C. albicans* development, but it did notably suppress hypha generation and hyphal prolongation *in vitro*. Furthermore, development suppression could occur in intralipid when replenished with capric acid, there is a fatty acid in SMOFlipid but no in intralipid. *C. albicans* biofilm generation in PN solutions was also found to be dependently inhibited by capric acid (Willems et al., 2019).

### Polysaccharide Inhibitors

Another newly discovered chitosan has been identified as a polysaccharide that inhibited *C. albicans* planktonic development (HMW, 1 mg/mL; LMW, 3 mg/mL) *in vitro*. With respect to biofilm development, chitosan suppressed *C. albicans* adherence (ca. 95%) and biofilm generation (> 90%) and decreased mature biofilms by ca. 65% and dual species biofilms (*C. albicans* and *S. mutans*) by ca. 70%. The outcomes demonstrate that this molecule is potential to be an anti-*Candida* agent working with *C. albicans* infections (Costa et al., 2014). Further, chitosan resistance, aggressive development, biofilm generation, and virulence in *C. albicans* require that MSS2 maintain mitochondrial function *in vitro* (Ke et al., 2021).

### Nanoparticles

Due to the weak penetration and non-specificity of antifungal and non-antifungal drugs, it is hard to treat biofilm generation. For addressing this issue, researchers are striving to augment the penetration of drugs into the extracellular matrix of biofilm. Over the past few years, several metal nanoparticles have become potential candidates in treating microbial infections because of their potential as effective antimicrobial agents. Hernandez-Delgadillo et al. (2013) found that aqueous colloidal bismuth oxide nanoparticles exhibited antimicrobial activity against *C. albicans* development (decreasing colony scale by 85%) and inhibited biofilm generation *in vitro* (Hernandez-Delgadillo et al., 2013). In another vitro and vivo study, sustained nitric oxide-releasing nanoparticles resulted in cell death in *C. albicans* yeast and hyphal cells, inhibiting biofilm generation *in vitro* and in a rodent central venous catheter model (Ahmadi et al., 2016).

### Other Inhibitors

RNA aptamers chosen against yeast cells suppress *C. albicans* biofilm generation *in vitro* (Bachtiar et al., 2019). Researchers screened three protease inhibitor libraries including 80 compounds for their inhibitive abilities against *C. albicans* biofilm formation *in vitro* and vivo and interference in mature biofilms. The outcomes demonstrate that through integrating normative antifungal agents with given protease inhibitors, it may be a therapy method to prevent and treat *C. albicans* biofilm infections (Lohse et al., 2020). Besides, it was observed that human serum weakens biofilm generation by preventing the adherence of *C. albicans* cells *in vitro*. This answer may relate to the downregulation of adherence-associated genes ALS1, ALS3, and BCR1. The administrative serum ingredient is protease-resistant and heat stable (Ding et al., 2014) (Table l).

**TABLE 1:** Inhibitors involved in *C. albicans* biofilm formation.

	Inhibitors	Biofilm-related processes inhibited	Research type	References
Natural product	Eucarobustol E	Hyphal formation	vitro	Liu et al., 2017
	CLEO, camphor, fenchyl alcohol	Hyphal formation	vitro	Manoharan et al., 2017
	purpurin	Hyphal formation and metabolic activity	vitro	Tsang et al., 2012
	5MI2CA and 1MI2CA	Hyphal formation	vitro and vivo	Lee et al., 2018
	7-benzyloxyindole	Hyphal formation	vitro and vivo	Manoharan et al., 2018
	HHQ	Unknown	vitro and vivo	Reen et al., 2016
	Riccardin D	Hyphal formation	vitro	Li et al., 2012
	Bisbibenzyls	Hyphal formation	vitro	Zhang et al., 2011
	Perillaldehyde	Hyphal formation	vitro and vivo	Chen et al., 2020
	Shikonin	Hyphal formation	vitro and vivo	Yan et al., 2019
Antifungal agents	doxycycline	Unknown	vitro	Miceli et al., 2009; Gao et al., 2013
	NSAIDs	Unknown	vitro	Alem and Douglas, 2004; de Quadros et al., 2011; Ells et al., 2011
	ibuprofen	Unknown	vitro	Arai et al., 2005; Ricardo et al., 2009
	Ambroxol	Unknown	vitro	Rene et al., 2014
	anidulafungin	Unknown	vitro	Katragkou et al., 2010
	D,L-2- hydroxyisocaproic acid	Hyphal formation	vitro	Nieminen et al., 2014
	clioquinol	Hyphal formation	vitro	You et al., 2020
	Farnesol	Hyphal formation and Decreased thickness	vitro	Xia et al., 2017
	thiazolidinedione-8	Adherence, Hyphal formation and metabolic activity	vitro	Feldman et al., 2014
	Phenylthiazole Small Molecule	Adherence and metabolic activity	vitro and vivo	Mohammad et al., 2019
	CD101	Adherence	vitro	Chandra and Ghannoum, 2018
	[C16MIM]Cl	Killing of biofilm cells	vitro	Reddy and Nancharaiah, 2020
	Toremifene	Unknown	vivo	De Cremer et al., 2014
	nitric-oxide releasing aspirin	Adherence and hyphal formation	vitro	Madariaga-Venegas et al., 2017
	Miltefosine	Dispersion	vitro	Vila et al., 2016
	caspofungin	Dispersion	vitro	Uppuluri et al., 2011
Microorganisms	Lactobacillus	Adherence and hyphal formation	vitro and vivo	Noverr and Huffnagle, 2004; Nguyen et al., 2011; dos Santos et al., 2019; MacAlpine et al., 2021; Zeise et al., 2021
	E. faecalis bacteriocin	Hyphal formation	Vitro and vivo	Graham et al., 2017
	Pseudomonas aeruginosa	Hyphal formation	vitro	Salvatori et al., 2020
	Streptococcus mutans	Hyphal formation	vitro and vivo	Barbosa et al., 2016
	diazaspiro-decane structural analogs	Hyphal formation	vitro and vivo	Pierce et al., 2015
Phenotypic screening				
	Trichoderonin, Nanaomycin, Rapamycin et al.	Hyphal formation	vitro	Pierce et al., 2014
	Anisomycin, V alinomycin, Bacitracin et al.	Unknown	vitro	Pierce et al., 2014
Protein and peptides inhibitors	Pc-CATH1, Cc-CATH2, Cc-CATH3 and cathelicidin-BF	Unknown	vitro and vivo	Yu et al., 2016
	LL-37, KE-18 and KR-12	Unknown	vitro	Luo et al., 2017
	TNF	metabolic activity and yeast morphology	vitro	Rocha et al., 2017
	QQ	Hyphal formation	vitro	Weiland-Brauer et al., 2019
	hLF1-11 peptide	metabolic activity and yeast morphology	vitro	Morici et al., 2016
	Decapeptide OSIP108	Unknown	vitro	Delattin et al., 2014
Lipid inhibitors	Cinnamaldehyde	Unknown	vitro	Khan et al., 2017
	Sophorolipid	Hyphal formation	vitro	Haque et al., 2016
	Smoflipid	Hyphal formation	vitro	Willems et al., 2019
Polysaccharide inhibitors	Chitosan	Adherence	vitro	Costa et al., 2014
	MSS2	Adherence	vitro	Ke et al., 2021
Nanoparticles	Bismuth oxide aqueous colloidal nanoparticles	Unknown	vitro and vivo	Hernandez-Delgadillo et al., 2013
	Nitric Oxide-Releasing Nanoparticles	metabolic activity	vitro and vivo	Ahmadi et al., 2016
Other inhibitors	RNA aptamers	Hyphal formation	vitro	Bachtiar et al., 2019
	Combination of Antifungal Drugs and Protease Inhibitors	Unknown	vitro	Lohse et al., 2020
	Human serum	Adherence	vitro	Ding et al., 2014

## Protecting the Intestinal Epithelial Barrier

### Mucus Layer

The mucus layer covering gastrointestinal mucosa is the first line of defense against invasions generating from luminal content. It chiefly consists of high molecular weight glycoproteins called mucins (MUC).

Current researches have identified butyric acid as a main source of energy for intestinal epithelial cells that can raise the mucus-layer supplement rate *in vitro* models and butyrate is able to upregulate colonic mucins at the transcriptional level and even better when it is the major energy source of the cells (Gaudier et al., 2004). Inflammatory responses in the gut can be retarded by this short-chain fatty acid through reducing the expression of INF-γ, TLR2, and TNF-α *in vitro* (Elce et al., 2017). Some researches have disclosed that there are more A. muciniphila-derived extracellular vesicles (AmEVs) in the fecal samples of healthy controls compared with those of patients with T2D. In addition, AmEV administration enhanced tight junction function, reduced body weight gain and improved glucose tolerance in high-fat diet (HFD)-induced diabetic mice. To test the direct effect of AmEVs on human epithelial cells, cultured Caco-2 cells were treated with these vesicles. AmEVs decreased the gut permeability of lipopolysaccharide-treated Caco-2 cells, whereas Escherichia coli-derived EVs had no significant effect. Interestingly, the expression of occludin was increased by AmEV treatment. Thus, *A. muciniphila* is capable of restoring mucus layer thickness, relieving intestinal inflammation reactions from pili-like proteins, and improving inflammation-caused obstacle integrity damage, thus decreasing gut barrier demolition (Chelakkot et al., 2018). *In vivo* model showed that ILC3-derived IL-22 can also induce expression of tissue protective mucins and antimicrobial peptides including RegIIIβ, RegIIIγ, S100A8 and S100A9 by acting on the intestinal epithelium (Sonnenberg et al., 2011) (Figure l).

**FIGURE 1:**
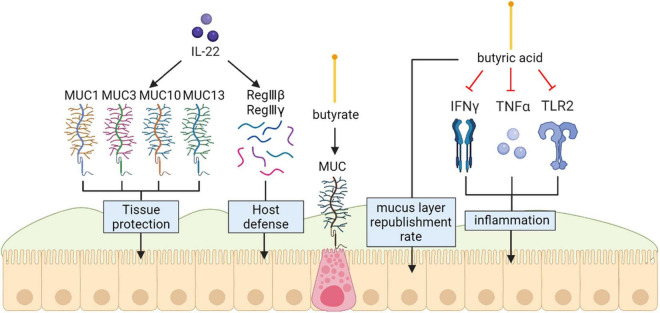
Mucins (MUC) protect the intestinal epithelial barrier. Exogenous IL-22 involved in producing protective mucus (MUC1, MUC3, MUC10 and MUC13) and induce expression of antimicrobial peptides including RegIIIβ, RegIIIγ. Butyrate specifically modulates MUC gene expression in intestinal epithelial goblet cells deprived of glucose Inflammatory responses in the gut can be retarded by butyrate through reducing the expression of INF-γ, TLR2, and TNF-α *in vitro*.

### Toll-Like Receptors

**FIGURE 2:**
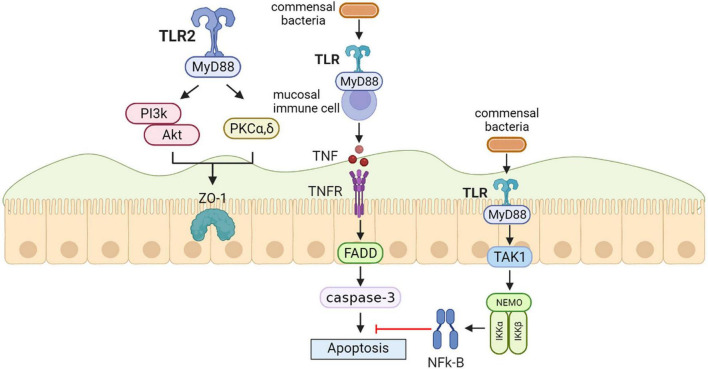
Toll-like receptors (TLRs) protect the intestinal epithelial barrier. In intestinal epithelial cells, TLR2 stimulation efficiently preserves zonula occludens-1 (ZO-1)-associated barrier integrity against stress-induced damage, which is controlled by positive signaling crosstalk between PI3K-Akt and conventional protein kinase C (PKC) isoforms *via* MyD88. In the steady-state conditio, commensal bacteria induce the production of physiologically optimal concentration of inflammatory cytokines [tumor necrosis factor (TNF)] in mucosal immune cells. Epithelial nuclear factor-κB (NFκB) has an important role in the anti-apoptotic function that is mediated by TAK1 and the inhibitor of NF-jB kinase (IKK) complex including NF-jB essential modulator (NEMO), IKKa, and IKKb.

Toll-like receptors (TLRs), innate immune sensors, exert a significant role in molding intestinal microbiota. TLRs may be deemed to an interface among the intestinal epithelial barrier, microbiota, and the immune system (Frosali et al., 2015). Furthermore, intestinal flora can mediate TLRs’ expression to maintain immune balance (Goto and Kiyono, 2012). TLR pathways are also closely associated with gastrointestinal barrier integrity and function; TLR2 and TLR4 paths are necessary for intestinal protection against acute mucosal injury by maintaining epithelial barrier integrity (Cario, 2008). The study suggests that the intestinal barrier offers places for intestinal flora that regulate TLRs’ immune reactions, particularly those in the intestine, because there are several immune cells and non-immune cells that contain TLRs in the intestinal barrier (de Medina et al., 2014). According to vitro, animal models and human results, TLR signaling in the intestinal epithelial cells notably increased the generation of IgA in the intestine. This role was regulated by TLR-caused expression of a given series of chemokines and cytokines that facilitated both the employment of B cells to the lamina propria and IgA class shift of B cells, thus decreasing intestinal inflammation (Shang et al., 2008). In a previous study, *B. fragilis*, through TLR2, induced cytokine generation and T cell differentiation. Unlike pathogens that trigger inflammatory responses through TLRs that result in immune responses to clear infections, symbiotic colonization by *B. fragilis* is actually enhanced by signaling *via* the TLR pathway promoting suppression of Th17 immunity. Thus, PSA evolved to engender host-bacterial mutualism by inducing mucosal tolerance through TLR2 activation of Treg cells (Round et al., 2011). Vitro and animal models also suggested the favorable impacts of *Yupingfeng* (YPF) are likely associated with stimulation of cytokines synthesis by triggering TLR2 and TLR4 paths, improving intestinal community structure and intestinal barrier integrity and functionality (Sun et al., 2016). A model of synergy was suggested whereby infection with *C. albicans* increases both the biomass of *S. oralis* and the TLR2 expression to critical levels required for mucosal proinflammatory signaling by this otherwise commensal organism. Whole mouse genome tongue microarray analysis showed that when compared with animals infected with one organism, the doubly infected animals had genes in the major categories of neutrophilic response/chemotaxis/inflammation significantly upregulated, indicative of an exaggerated inflammatory response. This response was dependent on TLR2 signaling since oral lesions, transcription of pro-inflammatory genes and neutrophil infiltration, were attenuated in TLR2-/-animals. Furthermore, *S. oralis* activated neutrophils in a TLR2-dependent manner *in vitro*. Thus, this study identifies a previously unrecognized pathogenic synergy between oral commensal bacteria (Xu et al., 2014). In intestinal epithelial cells, TLR2 stimulation efficiently preserves zonula occludens-1 (ZO-1)-associated barrier integrity against stress-induced damage, which is controlled by positive signaling crosstalk between PI3K-Akt and conventional protein kinase C (PKC) isoforms *via* MyD88. In parallel, the PI3K/Akt pathway limits proinflammatory TLR2-signaling through the Mapk-NFkB pathway (Cario, 2008) (Figure 2).

### Aryl-Hydrocarbon Receptor

**FIGURE 3:**
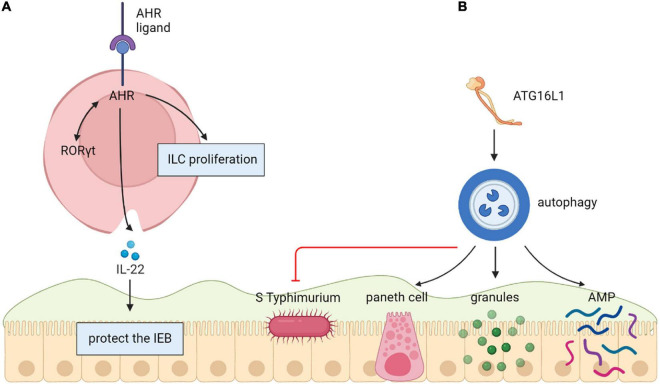
**(A)** Aryl-hydrocarbon receptor (AHR) protect the intestinal epithelial barrier. Directly binding to the Il22 promoter, AHR induces Il22 expression in ILC3 by acting together with RORγt. **(B)** Autophagy (ATG16L1) protects the intestinal epithelial barrier. The decrease of Atg16l1 led to fewer Paneth cells and abnormal granule morphology, resulting in decreased expression of AMP, which growing inflammation and comprehensive translocation of bacteria.

An aryl-hydrocarbon receptor (AHR) as a kind of toxin sensor binds to various endogenous and exogenous chemicals. In a previous study, AHR expression was increased in gut resident innate lymphoid cells (ILCs) (Jacquelot et al., 2019). The lack of AHR was related to decreased ILCs numbers and reduced ILC3-derived IL-22 *in vitro* and vivo (Kiss et al., 2012; Lee et al., 2012; Jacquelot et al., 2019). In another study, AHR directly bound to the Il22 promoter and had a synergistic effect on RORγt to promote Il22 expression in ILC3 (Qiu et al., 2012). *In vitro* and vivo studies have shown that the cytochrome P4501 (CYP1) family enzymes mediated the metabolic elimination of AHR ligands. Moreover, structural expression of CYP1 enzymes greatly decreased the usability of AHR ligands and resulted in the loss of gut ILC3 and Th17 cells (Schiering et al., 2017). Therefore, elementary Cyp1a expression or entire loss of AHR in mice improves their sensitivity to *C. rodentium* related to hindered IL-22 generation (Kiss et al., 2012; Qiu et al., 2012; Schiering et al., 2017). There are studies that emphasize how constant control of the availability of AHR ligands *via* intestinal epithelial cells offer vital feedback to immune cells, which thereby forms mucosal protections (Jacquelot et al., 2019) (Figure 3).

### ATG16L1

The autophagy gene ATG16L1 is related to protection from intestinal epithelial infection. A study showed that there were fewer Paneth cells and unusual granule morphology in Atg16l1f/f × Villin-cre mice, resulting in decreased AMP expression. Congruent with these defective immune responses, Atg16l1f/f × Villin-cre mice had improved inflammation and comprehensive bacterial translocation by comparing with control mice. The limitations of such in mouse model was that there is a compelling need for new therapeutic approaches to modulate specific pathways important in autoinflammatory and infectious diseases. Researchers tried to screen Modulators of autophagy to evaluate their effects on antibacterial responses in human epithelial cells. But few studies have been done on humans (Conway et al., 2013) (Figure 3).

### Cold-Inducible RNA-Binding Protein

**FIGURE 4:**
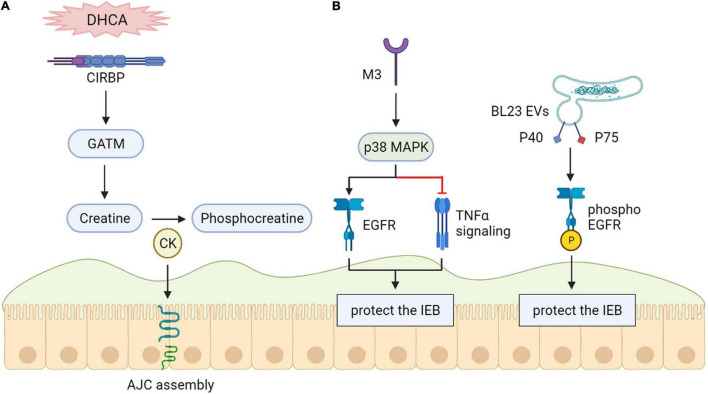
**(A)** Cold-inducible RNA-binding protein (CIRBP) protect the intestinal epithelial barrier. CIRBP is related to intestinal epithelial barrier maintenance during DHCA. **(B)** Epidermal growth factor receptors (EGFR) protect the intestinal epithelial barrier. The maintenance of epithelial barrier function may be helped by M3 receptor-induced activation of p38 MAPK through activation of EGFR. Phosphorylation of the epidermal element factor receptor (EGFR) can be induced by lactobacillus casei BL23 extracellular vesicles (BL23 EVs) because of proteins P40 and P75 that are bound to their surface.

Intestinal injuries occurring during deep hypothermic circulatory arrest (DHCA) are hazardous for clinical outcomes. Recent vitro and animals studies revealed that cold-inducible RNA-binding Protein (CIRBP) provides a protective effect in cases of hypothermia. These findings demonstrated the possibility of utilizing innate mechanisms of CIRBP to sustain the intestinal epithelial barrier during DHCA for the first time. This utilization will probably become a targeted treatment to prevent or relieve intestinal injury and relevant complications (Li et al., 2019) (Figure 4).

### Epidermal Growth Factor Receptors

Vitro research results demonstrate that M3 receptor-induced activation of p38 MAPK might maintain epithelial barrier function by downregulating TNF-α signaling and activation of Epidermal growth factor receptors (EGFR) instead of H1 (Uwada et al., 2017). Both P40 and P75 proteins were shown to show anti-apoptotic features containing EGFR phosphorylation and, in the case of P40, preventing the intestinal epithelium from triggered inflammation in a vitro model (Bauerl et al., 2020) (Figure 4).

### STAT3

Recently, researchers identified a subset of genes specially mediated by STAT3 in answer to leptin, particularly the TRIB1 and inhibitor of cytokine signaling 3 (SOCS3) genes, which have opposed effects in apoptosis control. Whole apoptotic genes were greatly accumulated in this gene set (*P* < 1Eμ05), boosting the assumption that protection results from leptin control of host apoptotic genes by STAT3.

**FIGURE 5:**
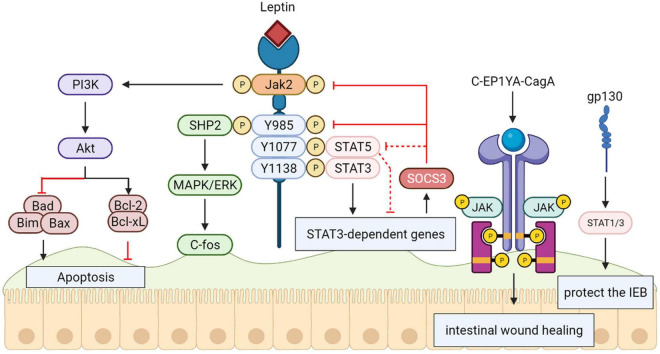
Signal transducers and activators of transcription 3 (STAT3) protect the intestinal epithelial barrier. The activation and tyrosine phosphorylation of Janus kinase 2 (JAK2) are resulted in by Leptin binding to the long form LRb, which causes follow-up phosphorylation of downstream tyrosine residues (Tyr985, Tyr1077, and Tyr1138) in the intracellular tail of LRb. The phosphorylation-dependent activation of signal transducers and activators of transcription 3 (STAT3) are bound and mediated by Phosphorylated Tyr1138, activating transcription of suppressor of cytokine signaling 3 (SOCS3) and other positive effectors of leptin action. The SH2-containing tyrosine phosphatase SHP-2 is recruited by Phosphorylated Tyr985, activating the signaling path culminating in extracellular signal-regulated kinase (ERK) activation. The C-EPIYA-CagA-mediated JAK/STAT Pathway and gp130-mediated STAT1/3 signaling plays a protective role in intestinal epithelium.

Interestingly, *in vivo* researches on amebiasis show that both SHP-2 and STAT3 are necessary for leptin-mediated protection (Guo et al., 2011), suggesting the need for a more sophisticated protective signaling mechanism during infection of the intestinal epithelium (Marie et al., 2012). Additionally, the C-EPIYA-CagA-mediated JAK/STAT pathway promoting cell migration IL6 has a significant effect on the initial stage of intestinal wound healing, and gp130-mediated STAT1/3 signaling plays a protective role in the intestinal epithelium in correlation with STAT3 function in epithelial migration during epidermal wound healing *in vitro* (Sano et al., 1999) (Figure 5).

### Lipopolysaccharide/CD14

**FIGURE 6:**
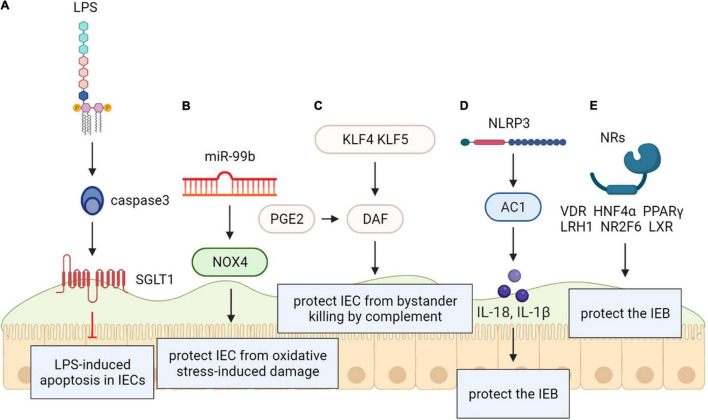
**(A)** Lipopolysaccharide (LPS/CD1) protects the intestinal epithelial barrier. SGLT-1-mediated glucose uptake may be activated by CD14 activation in intestinal epithelial cells, independently of TLR-4, for the protection of the epithelium against LPS-induced apoptosis. **(B)** NADPH oxidase 4 (NOX4) protects the intestinal epithelial barrier. The intestinal epithelium from oxidative stress-caused damage may be protected by MiR-99b-mediated NOX4 downregulation. **(C)** Krüppel-like factors (KLF) protect the intestinal epithelial barrier. Rapid protection against complementary attacks may be provided by synergistic induction of DAF by COX/PGE2and KLF4/5 after intestinal mucosal injury. **(D)** Recombinant NLR Family, Pyrin Domain Containing Protein 3 (NLRP3) protects the intestinal epithelial barrier. NLRP3 induced generation of IL-18 in intestinal epithelial cells can be protective. **(E)** Nuclear receptors (NRs) protect the intestinal epithelial barrier. Within the intestinal epithelium, NRs including VDR, HNF4α, LXR, PPARγ, LRH1, and NR2F6 exert protective effect on intestinal epithelial integrity.

Recent vitro model demonstrates that excess apoptosis and obstacle deficiencies triggered by Lipopolysaccharide (LPS) exposure are present *via* improved glucose uptake enterocytes. The reported research shows that a novel cell signaling path created by activating CD14 in intestinal epithelial cells, independently of TLR-4, may induce a SGLT-1-mediated glucose uptake to prevent the epithelium from LPS-caused apoptosis. This new pathway may help identify therapeutic targets in a variety of intestinal disorders (Yu et al., 2006) (Figure 6).

### NADPH Oxidase 4

Vitro and animals models reveal that NADPH oxidase 4 (NOX4) as an effective reactive oxygen species generator was considered a direct miR-99b target. Researchers speculated that protecting the intestinal epithelium from oxidative stress-induced injury may be related to miR-99b-mediated NOX4 downregulation (Chandra et al., 2015) (Figure 6).

### Krüppel-Like Factors

Vitro and animal models have documented the decay-promoting element (DAF) protects the intestinal mucosa from bystander killing by complement. Prostaglandin E2 (PGE2) promotes the expression of DAF that may prevent the tumor environment from complement attack. Krüppel-like factors (KLFs) are evolutionarily kept zinc finger-including transcription elements with various administrative functions in cell propagation and differentiation. DAF may have protective functions on both normal intestinal epithelium and intestinal neoplasia, so we can speculate that both KLF4 and KLF5 exert similar effects on regulating DAF expression. These novel findings provide insight into the functional role of the COX/PGE2system and KLF transcription factors in the gut and may contribute to new therapeutic strategies for a variety of intestinal disorders (Shao et al., 2008) (Figure 6).

### NLRP3

As an intracellular multiprotein signaling complex, Inflammasome is related to pathogen sensing and inflammatory response initiating in physiological and pathological states. The NLRP3 inflammasome, the most typical inflammasome, which has been identified as a sensor of cell stress that is strictly controlled in resting cells.

Nevertheless, changed management of the NLRP3 inflammasome is exhibited in many pathological states such as cancer and autoimmune diseases. It was proven that NLRP3 expression is post-transcriptionally regulated, and diversified miRNA have been involved in post transcriptional control of the inflammasome. Zaki et al. disclosed that NLRP3-induced generation of IL-18 in intestinal epithelial cells can be protective and leads to epithelium integrity in experimental colitis *in vitro*, animal models and human results. Therefore, the Nlrp3 inflammasome is critically involved in the maintenance of intestinal homeostasis and protection against colitis (Zaki et al., 2010; Tezcan et al., 2019) (Figure 6).

### Nuclear Receptors

Gastrointestinal (GI) homeostasis is strongly dependent on nuclear receptor (NR) functions. They play a variety of roles ranging from nutrient uptake, sensing of microbial metabolites, regulation of epithelial intestinal cell integrity to shaping of the intestinal immune cell repertoire. Several NRs are associated with GI pathologies; therefore, systematic analysis of NR biology, the underlying molecular mechanisms, and regulation of target genes can be expected to help greatly in uncovering the course of GI diseases. Within the intestinal epithelium, nuclear receptors (NRs) including VDR, HNF4α, LXR, PPARγ, LRH1, and NR2F6 have a protective effect on intestinal epithelial integrity (Klepsch et al., 2019); declined mRNAs have also been recognized in intestinal samples from IBD patients (Ahn et al., 2008; Li et al., 2015). PRAP1 has been identified an intrinsically disordered protein that is highly expressed by the gastrointestinal epithelium and roles on exposed surfaces to prevent the obstacle from oxidative insult *in vivo* (Wolfarth et al., 2020) (Figure 6).

## Conclusion

As fungal pathogens, *C. albicans* are known for their capacity to cause mucocutaneous and systemic infections in human hosts. Biofilm formation causes increasing *Candida* resistance to antifungal agents, which results in the failure of traditional antifungal agents’ therapeutic measures. Current advances on different natural product, antifungal agents, microorganisms, protein and peptides inhibitors, lipid inhibitors and polysaccharide inhibitors have made a growth of the knowledge of the intricate mechanism underlying the biofilm resistance. Recently, phenotypic Screening and different nanoparticles have also appeared as antibiofilm agents against *C. albicans* and gaining momentum. Yet, a limitation of this method is that most of the results from *in vitro* and animal models. If we want to apply the method of inhibiting *C. albicans* biofilms to clinical treatment, one challenge is that few studies have applied the results to human trials so that we cannot rule out whether other intestinal flora and their metabolites may have an effect on these biofilm inhibitors. On the other hand, new progress on molecules that protect the intestinal epithelial barrier like MUC, TLR, AHR, ATG16L1, CIRBP, EGFR, LPS/CD14, NOX4, STAT3, KLF, NLRP3 and Nuclear Receptors have improved the understanding of protective mechanism of intestinal epithelium barrier. However, these molecular studies did not specifically target intestinal infections of *C. albicans.* Therefore, further studies need to be carried out in order to validate the mechanism of how the intestinal barrier prevents *C. albicans* invasion. A key strength of the review is that the combination of preventing *C. albicans* from forming biofilms and using molecules to protect the intestinal epithelial barrier has not previously been reported for the treatment of *C. albicans* intestinal infection. Thus, the combination of these two methods is a novel idea to improve the therapeutic effect of *C. albicans* intestinal invasion. In terms of directions for future research, further work could focus on which combinations of antibiofilm and mucosal enhancement treatments were more effective in inhibiting *C. albicans* biofilm formation and intestinal infection and what are the mechanisms. We hope to develop effective strategies to treat *C. albicans* drug resistance and invasive intestinal infections in the near future.

## Author Contributions

Both authors listed have made a substantial, direct, and intellectual contribution to the work, and approved it for publication.

## Conflict of Interest

The authors declare that the research was conducted in the absence of any commercial or financial relationships that could be construed as a potential conflict of interest.

## Publisher’s Note

All claims expressed in this article are solely those of the authors and do not necessarily represent those of their affiliated organizations, or those of the publisher, the editors and the reviewers. Any product that may be evaluated in this article, or claim that may be made by its manufacturer, is not guaranteed or endorsed by the publisher.
